# Supplementary material for: A Global Screen for Assembly State Changes of the Mitotic Proteome by SEC-SWATH-MS
Source: Cell Syst. 2020 Feb 26;10(2):133–155.e6. doi: 10.1016/j.cels.2020.01.001 (PMC7042714; doi:10.1016/j.cels.2020.01.001)

Q9GZZ1 | NAA50_HUMAN | NAA50 MAK3 NAT13 NAT5
Monomer MW [kDa]: 19.398 Monomer expected elution fraction: 54

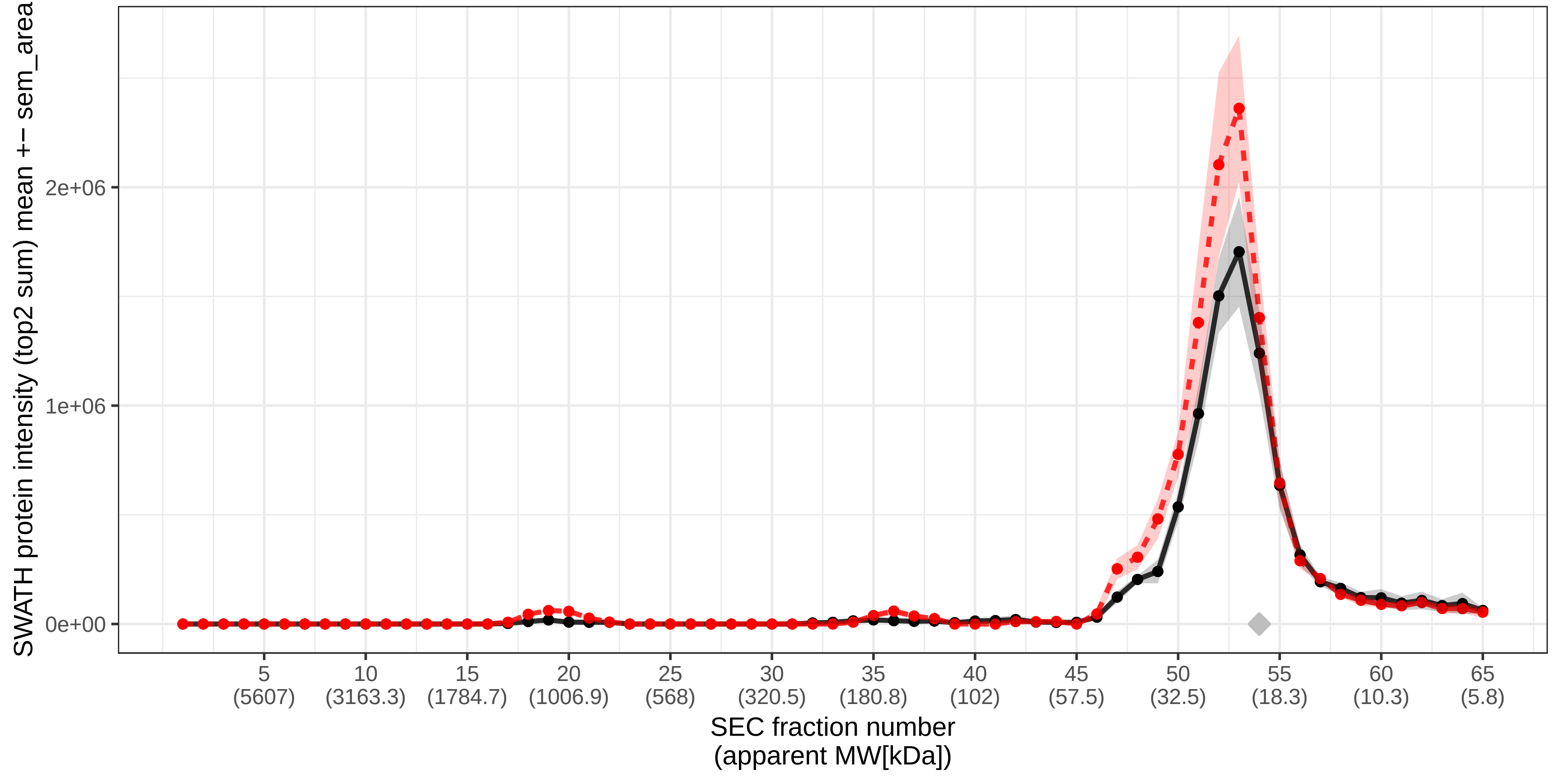

Supplement: Data S1. SEC-SWATH-MS Protein Chromatograms, Related to Figure 1 [file mmc6.zip › SECchrom_Q9GZZ1_NAA50_HUMAN_NAA50_MAK3_NAT13_NAT5.pdf]
